# Web-based tool for visualization of electric field distribution in deep-seated body structures and planning of electroporation-based treatments

**DOI:** 10.1186/1475-925X-14-S3-S4

**Published:** 2015-08-27

**Authors:** Marija Marčan, Denis Pavliha, Bor Kos, Tadeja Forjanič, Damijan Miklavčič

**Affiliations:** 1 University of Ljubljana, Faculty of Electrical Engineering, Trzaska 25, SI-1000 Ljubljana, Slovenia

**Keywords:** electroporation, electrochemotherapy, irreversible electroporation, electric field distribution, treatment planning, numerical modeling, web-based

## Abstract

**Background:**

Treatments based on electroporation are a new and promising approach to treating tumors, especially non-resectable ones. The success of the treatment is, however, heavily dependent on coverage of the entire tumor volume with a sufficiently high electric field. Ensuring complete coverage in the case of deep-seated tumors is not trivial and can in best way be ensured by patient-specific treatment planning. The basis of the treatment planning process consists of two complex tasks: medical image segmentation, and numerical modeling and optimization.

**Methods:**

In addition to previously developed segmentation algorithms for several tissues (human liver, hepatic vessels, bone tissue and canine brain) and the algorithms for numerical modeling and optimization of treatment parameters, we developed a web-based tool to facilitate the translation of the algorithms and their application in the clinic. The developed web-based tool automatically builds a 3D model of the target tissue from the medical images uploaded by the user and then uses this 3D model to optimize treatment parameters. The tool enables the user to validate the results of the automatic segmentation and make corrections if necessary before delivering the final treatment plan.

**Results:**

Evaluation of the tool was performed by five independent experts from four different institutions. During the evaluation, we gathered data concerning user experience and measured performance times for different components of the tool. Both user reports and performance times show significant reduction in treatment-planning complexity and time-consumption from 1-2 days to a few hours.

**Conclusions:**

The presented web-based tool is intended to facilitate the treatment planning process and reduce the time needed for it. It is crucial for facilitating expansion of electroporation-based treatments in the clinic and ensuring reliable treatment for the patients. The additional value of the tool is the possibility of easy upgrade and integration of modules with new functionalities as they are developed.

## Background

Exposing a biological cell to an electric field results in structural changes in the cell plasma membrane. If the electric field is sufficiently high, the changes in the trans-membrane voltage cause the membrane to become permeable to molecules which otherwise cannot cross it. The described phenomenon is termed electroporation [1] and can be either reversible or irreversible. If the electric field at given pulse characteristics exceeds the reversible electroporation threshold, the cell eventually returns to its normal state and survives; this is called reversible electroporation. However, if the electric field exceeds the threshold of irreversible electroporation or the cell is exposed to the electric field too long, the changes in the membrane lead to irreversible electroporation (IRE) characterized by cell death.

Both reversible and irreversible electroporation have found their use in treatment of tumors [2]. Combination of reversible electroporation and cytotoxic drugs used in chemotherapy produces an increased drug uptake in the tumor cells and a more efficient therapy. Such treatment has been termed electrochemotherapy (ECT) [3-5]. On the other hand, the permanent damage to cells caused by irreversible electroporation can be used to ablate the tumor cells directly. Since ablation with irreversible electroporation, for most part, does not employ a thermal mechanism of cell death, as opposed to e.g. radiofrequency ablation, IRE has also been termed non thermal irreversible electroporation (NTIRE) [6-8]. The basis of both therapies is the application of electric pulses with adequate amplitude, duration and pulse repetition frequency using specially designed pulse generators and electrodes [9].

While the ECT for skin tumors has already been accepted into several clinical guidelines, the application of ECT and IRE on deep-seated tumors is still in an earlier clinical phase of testing [5,10-14]. In the case of deep seated tumors, it is challenging to ensure the complete target volume coverage with a sufficiently high electric field, which is the main prerequisite for successful tumor treatment [15-17]. Positions and the trajectory of electrode insertion have to be precisely determined along with optimal pulse parameters. Taking into account the additional problem of patient and tumor anatomical variability, the best way to ensure that the target tissue is exposed to sufficiently high electric fields is by performing patient-specific treatment planning [18,19].

In order to improve the prediction of treatment outcome, we have developed a web-based tool for visualization of electric field distribution on geometric model of patient anatomy acquired through segmentation of medical images. As an inspiration for our workflow, we have used the radiotherapy and radiofrequency ablation treatment planning (TP) procedures, which consist of several steps [19]: medical imaging of the patient, image processing and extraction of model geometry, and numerical modeling with determination of the optimal treatment parameters. All these steps have been adjusted for electroporation-based treatment planning.

Our aim was to create a tool which the clinicians could easily use without the help of engineers or without deep technical knowledge. Such a tool should relieve the user from performing unnecessary steps from the engineering domain while keeping the necessary level of robustness and reliability. In this way the tool aids the spread of use of ECT and IRE to clinics with no or minimal specific engineering support. The final product is a web-based tool for visualization of electric field distribution and planning of electroporation-based treatments which is described in detail in following sections.

## Methods

The developed workflow for electric field visualization consists of several steps. The user first uploads medical images in DICOM format or selects such images from a previously-uploaded case. After uploading, there is an option for immediate and permanent anonymization of the data. Medical images can, then, be segmented manually or automatically by selecting the desired segmentation target (e.g. liver on MRI images, or bone tissue on CT images, etc.). It is important to note that, in case of tumor segmentation, it can currently be done only using the manual segmentation module.

**Figure 1:**
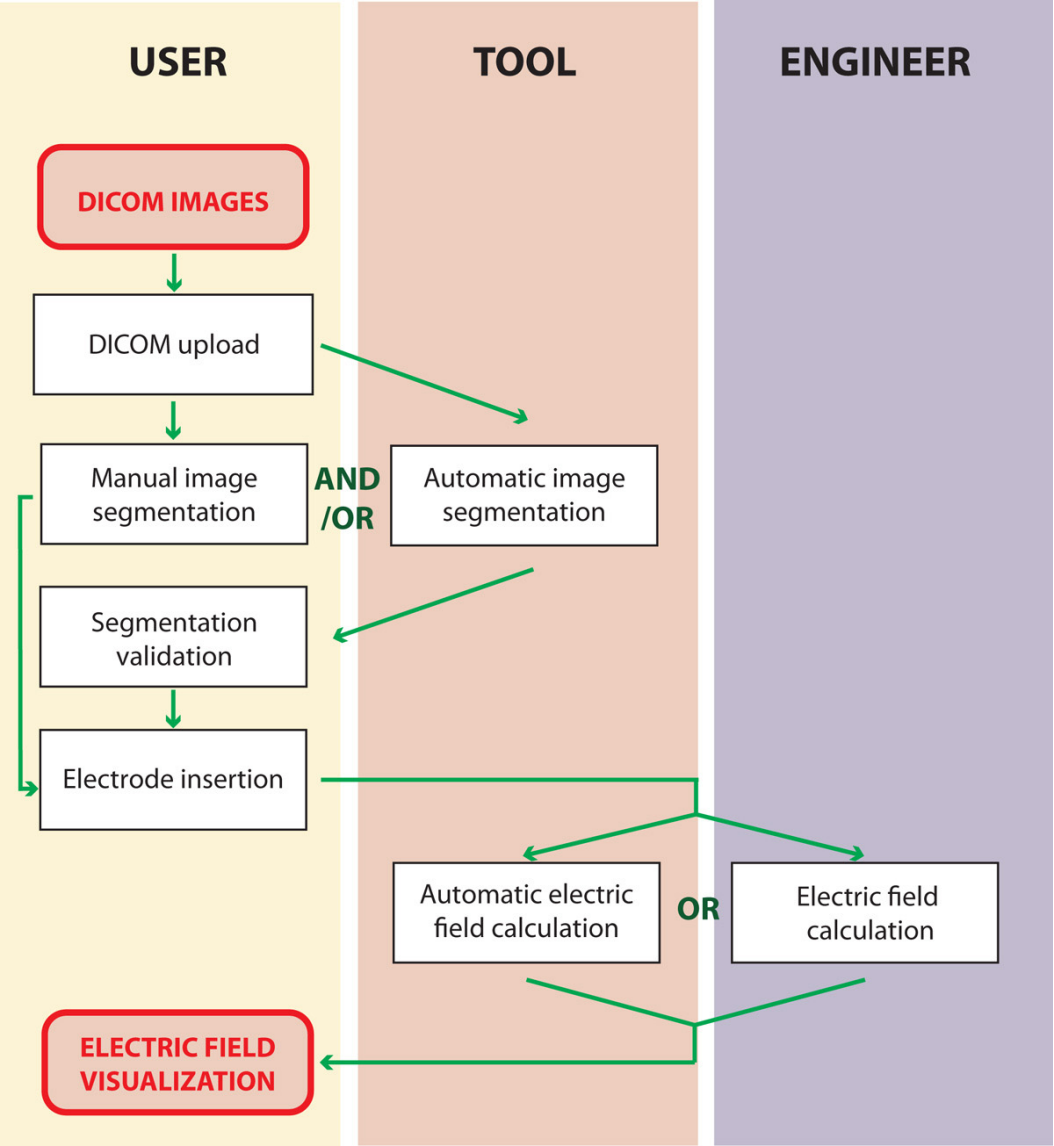
**Diagram of the workflow of the web-based tool for visualization of the electric field distribution**.

**Figure 2:**
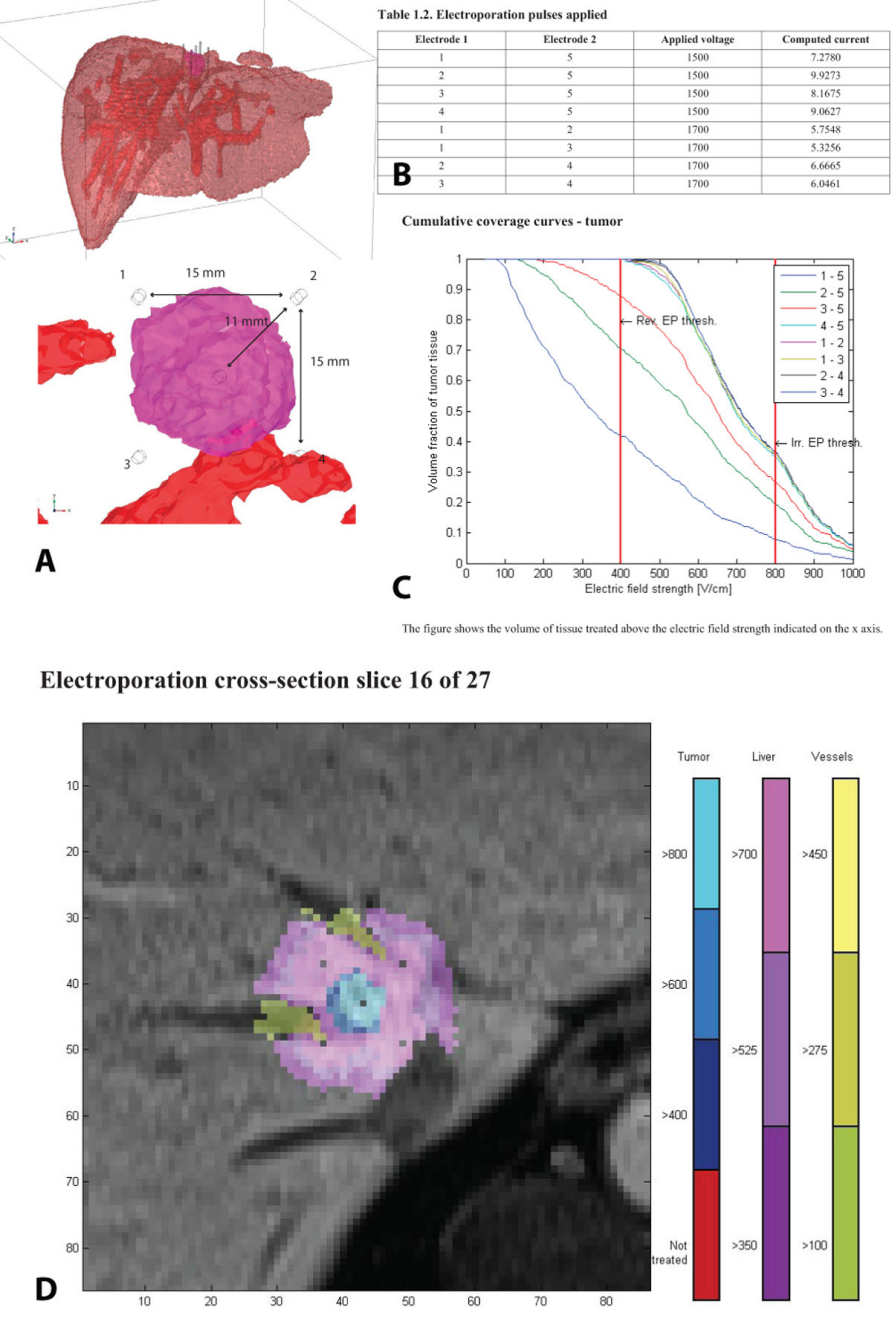
**An example of a treatment report file for ECT of liver**. A. 3D model of the case with marked positions of electrodes. B. Table containing optimal voltages per electrode pair. C. Cumulative coverage curves for the tumor tissue. D. Electric field distribution overlaid on original patient images

After automatic segmentation, the user is given the possibility to inspect and correct the obtained segmentation results and validate them. Finally, the user specifies the trajectory vector of the inserted electrodes and either decides to execute automatic electric field modeling or to send the segmented case to an experienced engineer from our team to evaluate and model the case. In either case, the final output that is given to the user is a 'Treatment report' in the form of a pdf file. This file contains all the information regarding electrode positioning, the optimal pulses that should be applied per each electrode pair, volumes of reversibly and irreversibly treated areas of all tissues and 2D illustrations of the electric field distribution. The diagram of the workflow of our tool is shown in Figure 1, while an example treatment plan is shown in Figure 2.

### Medical image segmentation

The process of image segmentation depends on the target organ. So far, we have implemented algorithms for liver and bone tissue segmentation of human patients and brain tissue segmentation of canine patients, therefore making the tool available also for veterinary medicine [20-24].

Automatic liver segmentation using our tool can be performed on MRI or CT images. Methods for liver extraction using MRI data are based on region growing, adaptive thresholding and active contours according to the work of Pavliha et al. [25]. A different, two-stage method is used for CT liver extraction: rough estimation of liver region by identifying the largest connected component on Euclidean distance maps is followed by precise determination of liver boundary with active contours.

After liver tissue extraction is performed, the resulting liver geometry is used as a mask for hepatic vessels segmentation. The method for segmentation of hepatic vessels in both MRI and CT images is based on vesselness filtering and local thresholding in the neighborhood of the results of vesselness filtering [26]. The vesselness filter applied is based on the work of Frangi et al. [27] and can be easily adapted to detect vessels in both CT and MRI images.

The algorithms for canine brain extraction from T1 and T2 weighted MRI images are based on morphological operations and they consist of the following steps. First, the central slice of the brain is identified by connected component analysis of eroded images. The brain tissue from the remaining slices is extracted using the similarities of adjacent slices [28,29].

Bone tissue is extracted from CT images using thresholding-based method. All of the implemented algorithms for segmentation of different objects of interest work automatically and require no user input. The modular design of the tool allows additional methods to be easily added after being developed and tested. The objects that are not planned to be automatically extracted are the tumors, due to their high variability of shape, size and location. For this purpose, we have provided a tool for manual segmentation based on drawing contours by placing points on the original slices.

### Segmentation validation

The results of automatic segmentation of objects of interest are displayed to the user in the form of 2D contours overlaid over the original image slices. In order to ensure robustness and quality of the model extraction from medical images, the user is asked to validate results of automatic segmentation. In this phase, the user is able to manually change the segmentation results if needed.

Correction of individual object boundaries is possible by freely moving contour points to the desired position. For this purpose, the 2D contours of segmented objects are simplified and initially reduced to 20% of the original amount of points in the contour. The reduction of contour points is based on the measure of influence of a certain contour point which is calculated based on an angle and length between adjacent edges of the contour [30]. The number of points in the 2D contour, expressed as a percentage of the original number of contour points, can also be reduced or increased by the user on the level of an individual slice during the validation process itself. When the user chooses to change the number (percentage) of points in the contour, the new points are determined based on the reduction of the original contour as described above.

In case the user has already moved some points in space prior to choosing to change the number of contour points, the changed part of the contour is first reconstructed by fitting a piecewise cubic interpolation curve through the modified points. The reduction algorithm is then performed on the contour which consists of the original points that have not been changed and the points of the fitted curve. In case there is a complete object missing from the segmentation or the user deems adjusting the segmentation results by moving the contour points would be too laborious, he/she also has an option of erasing the segmentation results on a slice level and manually drawing the contour.

The manual drawing is supported by the manual segmentation tool described in the previous section which is also embedded in the module for manual validation.

### Electrode positioning

The validated model of the target organ with tumor and other structures of interest also requires a 3D model of electrodes added before numerical modeling can be performed. The size and type of the electrodes is pre-defined by the manufacturer while the treatment as being currently performed requires them to be parallel. The user thus needs to select the type of the commercially available electrodes, the length of the active part of the electrode, and the desired trajectory of electrode entry which will be used during the actual treatment.

**Figure 3:**
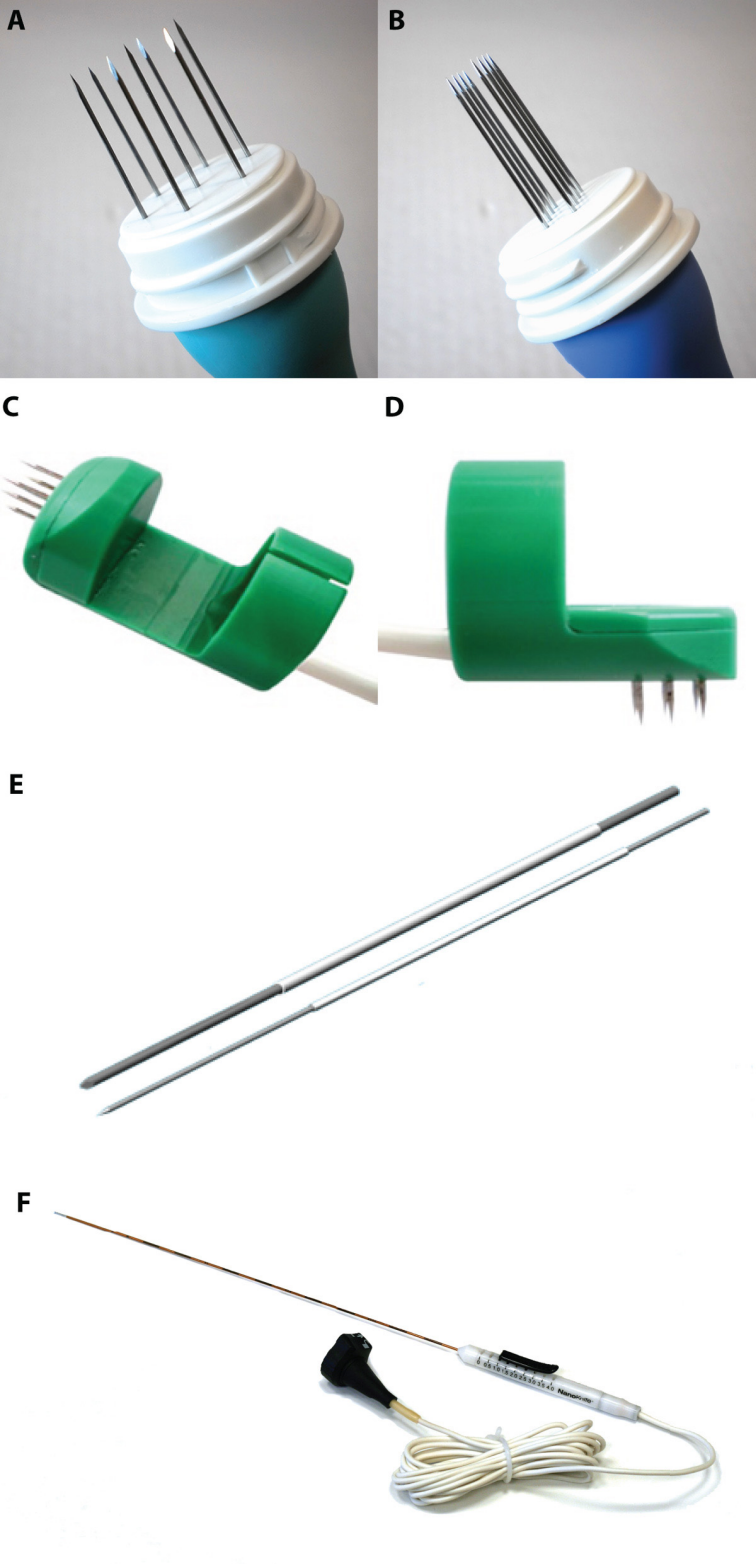
**Commercially available electrodes**. A. Hexagonal needle electrodes. B. Linear needle electrodes. C. Finger electrodes with axial needles. D. Finger electrode with perpendicular needles. E. Variable-geometry needle electrodes. F. Angiodynamics Nanoknife Variable geometry needle electrodes. All electrodes pictured in A-E are available from IGEA SrI.

The commercial electrodes that are currently supported by the tool are shown in Figure 3. The choice of the entry trajectory is made by placing a starting point and an ending point in two arbitrary 2D image slices of the patient. During the definition of the electrode entry trajectory, the user is aided by the visualization of nearby structures that limit electrode access, such as large vessels in the liver or bones in the case of head and neck tumors.

### Numerical modeling

Numerical modeling is currently performed using Comsol Multiphysics (Comsol AB, Stockholm, Sweden). Live link for Matlab provides an interface for Matlab and allow for complete control of the finite element method model setup and solving. To this end, we have developed code in Matlab which uses the segmentation provided by the previous steps to automatically build a model with all segmented tissues.

Electrodes are inserted into the model based on the positions on medical images specified by the user in the 'electrode positioning' module. Boundary conditions for voltage are then set on successive pairs of electrodes. For implementing different conductivity values and changing of conductivity due to electroporation [31,32], we use interpolation functions to specify the conductivity in each point of the model [33]. In this way, Matlab code allows to increase the conductivity value of the tissues in each point of the model as a function of the local electric field [31].

For mesh generation built-in meshing capabilities of Comsol Multiphysics are used. Since the model consists only of the electrodes and the bounding box of the region of interests, with the tissue parameters being handled by Matlab via lookup tables, the built-in free tetrahedral meshing algorithms have proven effective and robust. The electric field is computed iteratively until there is no further increase in conductivity. After voltages on all electrode pairs are computed, the total coverage of the target tissue and volumes of surrounding tissues covered with electric fields above the irreversible threshold are determined. Electric field distribution is also overlaid over the original medical images for easier visual representation [34].

It is important to note that the model sets the target electroporation thresholds according to pulse parameters (pulse number, duration, repetition frequency) that are most often used as standard settings in clinical applications. For ECT this means 8 pulses of 100 microsecond duration with repetition frequency of 1 Hz for and for IRE it is 90 pulses of 100 microsecond duration with repetition frequency of 1 Hz. In the case the user would like the pulse parameters to be different from these this issue can be resolved by direct contacting of the engineer via info@visifield.com.

## Results and discussion

### Web tool framework

**Figure 4:**
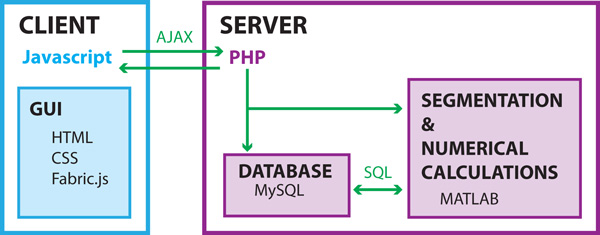
**Schematical representation of the web tool framework with main components and technologies used in their realization**.

The whole treatment planning procedure is embedded in a web-based client-server solution. The web page acts as a client graphical user interface. All of the segmentation algorithms along with numerical modeling are performed on a dedicated server running Matlab as the back-end engine. The results of the segmentation and numerical modeling along with original medical images are stored in a MySQL database and forwarded to the client side. The architecture of our web tool framework is presented schematically in Figure 4.

On the client side, Hyper-Text Markup Language (HTML) and Cascaded Stylesheets (CSS) are used for visualization of the content (i.e. Graphical User Interface - GUI), while JavaScript (JS) is used for client-side dynamic scripting. The main part of the server side is realized using the Hypertext Preprocessor (PHP) which acts as the content back-end processor and for executing server-side processes. Also, MATLAB is currently used on the server side as the main processing back-end engine for procedures. Structured-Query Language (MySQL) is used as the database containing all the required data tables, and for indirect communication between the GUI and MATLAB (i.e. some dedicated tables are used for interaction between different software parts).

Additionally, some open-source publicly available JS libraries that are used are FABRIC.JS and jQuery. FABRIC.JS is used for client-side graphics in the manual segmentation and validation modules. jQuery is used for client-side dynamic scripting, and for performing Asynchronous JavaScript and XML (AJAX) calls from the GUI to the PHP back-end.

### Graphical user interface

**Figure 5:**
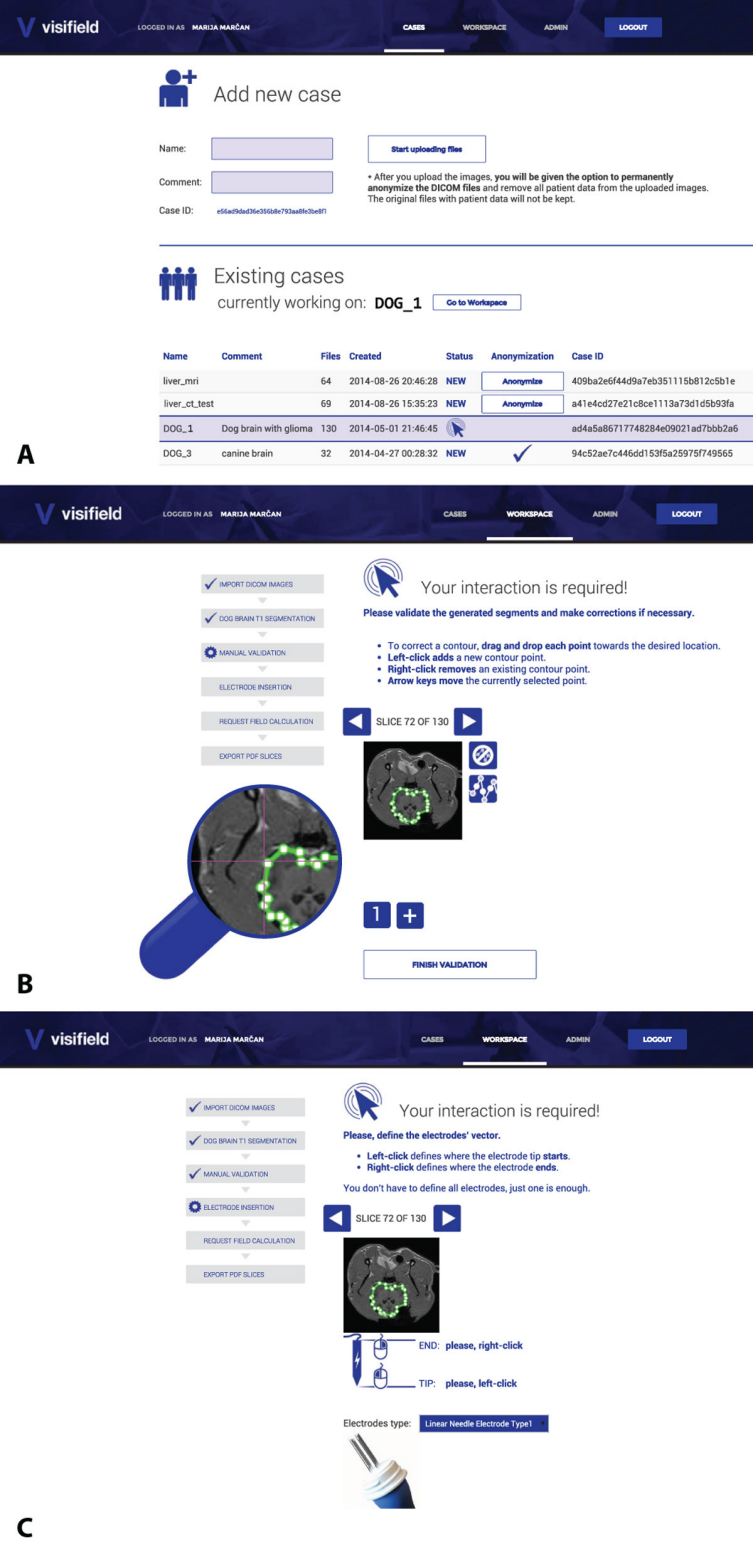
**The main working screens of the application**. A. Cases (i.e. uploading images and selecting the active patient). B. Workspace (i.e. selecting the procedure, validating segmentation results, downloading final results, etc.). C. Electrode insertion module, in which electrode type and entry point can be specified.

The tool is divided in two main working screens, shown in Figure 5: The first screen 'Cases' (Figure 5.A) permits adding new patients by uploading the DICOM images (by either dragging and dropping them into a rectangle on the screen, or by opening the classic file dialog). Existing patients are listed in the table and the currently selected patient (which is, then, available to work with in the Workspace screen) is marked. The 'Workspace' screen contains all the procedures and results of the currently selected patient. First, the user can select one of the predefined procedures (e.g. Electric field visualization in the Liver on CT images) or can create a customized own procedure (e.g. Import DICOM Images, Automatically segment bone tissue, Validate bone tissue, Insert electrodes, Request electric field calculation, Download PDF) by dragging and dropping these individual modules to form the desired custom procedure order.

The most advanced part of the 'Workspace' is the manual segmentation/validation module (Figure 5.B), which is invoked after the automatic segmentation (or can replace it for tissues that are not yet supported by any of the automatic segmentation modules). The manual segmentation/validation module allows displaying the results of automatic segmentation (which are 2D surfaces on slices) as closed contours consisting of several points. The user can rearrange these points in order to correct any possible mistakes made by the automatic segmentation. If any objects are missed, the user can easily add new or remove existing objects by clicking on the corresponding buttons below the currently-displayed slice. Likewise, if the number of the displayed contour points is inadequate, the user can perform point refinement on the current slice and instantaneously change the number of points the back-end had generated. Zooming is also possible and the zoom (displayed as the lens next to the currently-displayed slice) is dynamically updated as the mouse moves in order to allow a precise and easily-achievable accurate segmentation/validation on the original images.

The GUI for the electrode insertion module is shown in Figure 5.C. The user can pick one of the commercially available electrodes from the drop down menu. After the choice of the electrode type has been made the user is required to specify the entry trajectory of the electrodes. This is done by marking two points in any of the two patient image slices or possibly even in the same slice. Left mouse click places the starting tip of the electrode while the right mouse click places the end point of the electrode. The described procedure needs to be done only once regardless of the final electrode number, even in cases where several individual electrodes are needed as this number will be determined by numerical modeling as the electrodes are considered to be parallel to each other.

### Performance evaluation

Five independent users from four different institutions in different countries were given access to the web tool for evaluation purposes [35]. The users were not given any specific task, they were simply asked to use the tool according to their respective areas of interest. All five users have experience in medical image processing and electroporation-based treatments. The users were asked to provide feedback regarding the usage experience while we measured the times of performance of different tool components during their use. All of the users expressed the opinion that such a tool provides significant simplification of the treatment planning process, which they previously had to perform manually using different programs, taking up to 1-2 days of intense work.

The performance times of different tool components measured during evaluation by the five users are presented in Table 1, along with the level of user interaction required per individual component. The same table also provides information about which tissue types and imaging modalities each user has tested as well as the interaction type employed for each tissue/user.

**Table 1:** Performance times of different tool components during user evaluation of the tool and level of user interaction.

Component	Level of interaction	Avg. time - User 1	Avg. time - User 2	Avg. time - User 3	Avg. time - User 4	Avg. time - User 5
Image upload	Interaction required	29.5 s	177 s	13.5 s	85 s	7.5 s

Automatic segmentation	Automatic	N/A	59 s	25 min	N/A	50.3 s

Manual segmentation	Interaction required	14 min	N/A	5 min	5 min	13 min

Manual validation	Interaction required	N/A	10 min	N/A	N/A	53 min

Tissue type / Imaging modality / Interaction level		liver tumor / MRI / manual	pelvic bone / CT / automatic	1) liver / CT / automatic; 2) prostate / MRI / manual	bone / CT / manual	1) canine brain / MRI / automatic; 2) tumor / MRI / manual

## Conclusions

We have developed a web-based tool which embeds specifically-developed algorithms for medical image segmentation and numerical modeling and optimization, all for the purpose of generating patient-specific treatment plans for electroporation-based treatments. The need for treatment planning in electroporation-based treatments has already been recognized as a necessity [13,36-38]. Since patient-specific treatment planning is not a trivial task, a tool such as the one we have presented is necessary in order to enable routine clinical use of electroporation-based treatments. So far the implemented automatic segmentation algorithms are limited to human liver and hepatic vessels, bone tissue segmentation and canine brain tissue segmentation. However, other tissue types can be segmented using the manual segmentation module. Also, it is important to note that the development of the presented tool is an iterative process and its modular design allows easy upgrade and inclusion of new algorithms for automatic segmentation according to needs of the clinical community.

In contrast to radiotherapy, electroporation-based treatments are not yet supported in medical institutions by dedicated teams of biomedical engineers or medical physicists with necessary knowledge and experience in electroporation who could prepare treatment plans. Our solution removes the burden of complicated engineering procedures from the end-user while the minimum required amount of interaction ensures robustness and validity.

Additional value of the presented solution is that it is web-based with all of the computationally intensive tasks performed on a dedicated server we provide. This concept eliminates the need to install new programs on the end-user's computer, as opposed to majority of tools for medical image segmentation that are available today. Moreover, the user can access his or her own patients and treatment plans from any computer as all the data are stored on the server. The brief yet valued evaluation by experts in the field of image processing and electroporation-based treatments has shown that the tool significantly shortens the time necessary to generate a treatment plan, from 1-2 days to a few hours. Such advance can greatly help the expansion of electroporation-based treatments in the clinic and improve reliable treatment performance.

## Competing interests

The authors declare that they have no competing interests.

## Authors' contributions

MM designed and implemented the algorithms for hepatic vessel segmentation and segmentation validation, and wrote the paper. DP designed and implemented the algorithms for liver MRI segmentation, implemented the entire web framework of the tool, and helped in writing the paper. BK programmed the algorithms for numerical modeling, helped optimize database access, and helped writing the paper. TF designed and implemented the algorithms for liver CT and dog brain segmentation, and helped writing the paper. DM participated in conceiving the ideas for the tool, guided the research process and critically revised and directed the manuscript. All authors read and approved the final version of the manuscript.
